# Progression of ductal carcinoma in situ to invasive breast cancer: comparative genomic sequencing

**DOI:** 10.1007/s00428-018-2463-5

**Published:** 2018-10-04

**Authors:** S. C. Doebar, N. M. Krol, R. van Marion, R. W. W. Brouwer, W. F. J. van Ijcken, J. M. Martens, W. N. M. Dinjens, C. H. M. van Deurzen

**Affiliations:** 1 Department of Pathology, Erasmus MC Cancer Institute, Rotterdam, Netherlands; 2 Erasmus MC Cancer Institute, PO BOX 2040, 3000 Rotterdam, CA Netherlands; 3 Biomics, Erasmus MC Cancer Institute, Rotterdam, Netherlands; 4 Department of Medical Oncology and Cancer Genomics Netherlands, Erasmus MC Cancer Institute, Rotterdam, Netherlands

**Keywords:** Breast, Ductal carcinoma in situ, Progression, Genomics

## Abstract

Several models have been described as potential mechanisms for the progression of ductal carcinoma in situ (DCIS) to invasive breast cancer (IBC). The aim of our study was to increase our understanding of DCIS progression by using massive parallel sequencing of synchronous DCIS and IBC. We included patients with synchronous DCIS and IBC (*n* = 4). Initially, IBC and normal tissue were subjected to whole exome sequencing. Subsequently, targeted sequencing was performed to validate those tumor-specific variants identified by whole exome sequencing. Finally, we analyzed whether those specific variants of the invasive component were also present in the DCIS component. There was a high genomic concordance between synchronous DCIS and IBC (52 out of 92 mutations were present in both components). However, the remaining mutations (40 out of 92) were restricted to the invasive component. The proportion of tumor cells with these mutations was higher in the invasive component compared to the DCIS component in a subset of patients. Our findings support the theory that the progression from DCIS to IBC could be driven by the selection of subclones with specific genetic aberrations. This knowledge improves our understanding of DCIS progression, which may lead to the identification of potential markers of progression and novel therapeutic targets in order to develop a more personalized treatment of patients with DCIS.

Ductal carcinoma in situ (DCIS) is a non-obligate precursor of invasive breast cancer (IBC). However, no reliable biomarkers or clinical tests are available to predict which DCIS cases are most likely to progress. In-depth genetic studies of DCIS and synchronous IBC reported intra-tumoral genetic heterogeneity and genetic differences between DCIS and synchronous IBC [1, 2]. Based on these findings, an evolutionary bottleneck selection model has been proposed [3, 4]. According to this theory, distinct subclones with specific genetic changes are selected during the transition from DCIS to invasive disease. This leads to differences in the prevalence of specific mutations between the neoplastic DCIS cells and invasive counterpart [3, 5, 6]. In contrast to this model, a multiclonal evolution theory has been proposed, which assumes that multiple subclones in DCIS co-migrate during the transition from DCIS to IBC [4, 7]. To increase our understanding of DCIS progression, we performed massive parallel sequencing of synchronous DCIS and IBC. We reported overlapping mutations between synchronous DCIS and IBC combined with the presence of invasive-specific mutations, which support the theory that the progression from DCIS to IBC could be driven by the selection of subclones with specific genetic aberrations.

We examined the exomes of four patients diagnosed with estrogen receptor (ER) positive, human epidermal growth factor receptor 2 (HER2) negative DCIS, and synchronous IBC after surgical excision. All cases had an invasive ductal carcinoma that was graded in each case as grade 3. Regarding the in situ component, the DCIS grade was in all cases concordant with the grade of the invasive carcinoma. The proportion of DCIS in each case was ranging from 2 to 5 cm.

Figure 1 provides an overview of the workflow. Initially, fresh frozen (FF) tissue of IBC and normal cells were subjected to whole exome sequencing. The sequence reads were aligned to the human genome build 19 (hg19) using BWA [8]. For each sample, at least four gigabases of sequences were aligned to the genome with an average coverage of at least 120× for IBC and at least 70× for normal tissue samples. Subsequently, the aligned reads were processed using the Indel Realigner, Mark Duplicates, and PHRED Recalibration tools from the Genome Analysis Toolkit (GATK) [9] to remove systematic biases and to recalibrate the PHRED quality scores in the alignments. Genetic variants were called using the Unified Genotyper Tool from GATK.

**Fig. 1:**
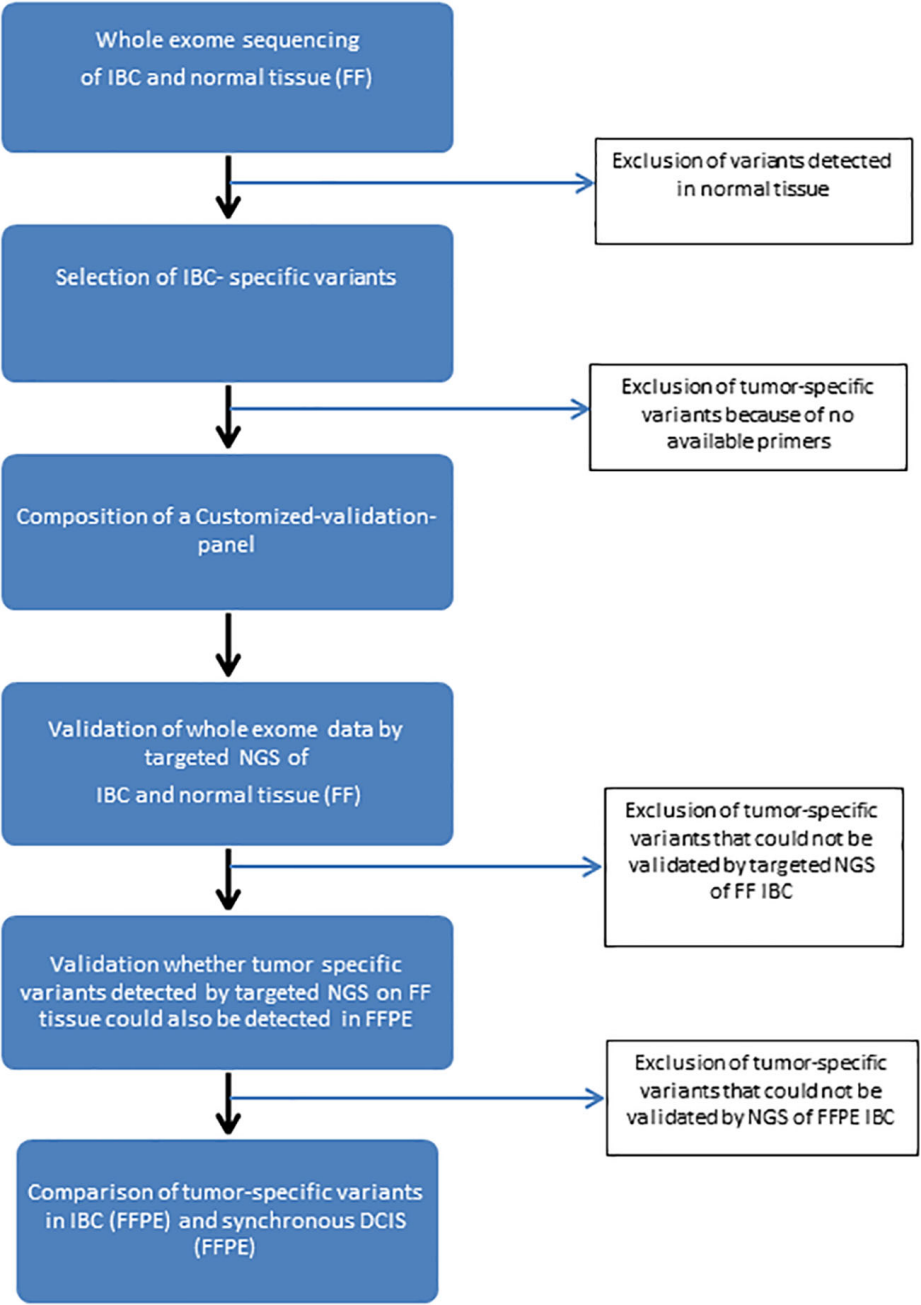
Schematic overview of the DNA-sequencing process from whole exome sequencing to targeted NGS

Based on the selected invasive tumor-specific variants, a specific custom-made panel was designed per patient. This custom-made cancer panel was performed on the Ion Torrent Personal Genome Machine (PGM) in order to validate whether those tumor-specific variants identified by whole exome sequencing could also be detected by targeted sequencing, using the same FF DNA samples, to ensure an accurate concordance between these two platforms. Subsequently, IBC-specific variants verified by Ion Torrent PGM were validated in DNA of formalin-fixed paraffin-embedded (FFPE) tissue of IBC, using a minimal genomic DNA input of 10 ng. In the final step, we validated only those IBC-specific variants verified in both FF tissue and FFPE tissue of IBC on DNA extracted from FFPE tissue of DCIS.

Library and template preparations were performed consecutively with the AmpliSeq Library Kit 2.0-384 LV and the Ion PGM Hi-Q Chef Kit. Templates were sequenced using the Ion PGM Hi-Q Chef Kit on an Ion 318v2 chip. Sequence information was analyzed with Variant Caller v4.4.2.1 (Life Technologies Carlsbad, CA, USA), and variants were annotated in a local Galaxy pipeline using ANNOVAR [10]. Variants were called when the position was covered at least 100 times. Non-synonymous somatic point mutations, insertions, and deletions that change the protein amino acids sequence and splice site alterations were selected. Variants found in at least 5% of the called reads and ≥ 10 variant-reads were considered reliable.

Based on whole exome sequencing of the four IBC samples, a total number of 792 tumor-specific variants were identified. Out of these 792 tumor-specific variants, primers were available for 585 variants. In total, 433 out of 585 tumor-specific variants could not be verified as a tumor-specific variant at the (Ion Torrent) validation stage in FF tissue of IBC. Out of the remaining 152 tumor-specific variants, 60 variants could not be validated in FFPE tissue of IBC. These variants were excluded for further analysis.

This resulted in a total number of 92 tumor-specific variants that remained for targeted validation in the DCIS component. Within each patient, a proportion of tumor-specific variants overlapped between the DCIS component and the invasive counterpart (in total 52 out of 92). In patient 1, all tumor-specific variants (17 out of 17) that were identified in IBC were also detected in the DCIS component. In the remaining three patients, the number of tumor-specific variants detected in the DCIS component was lower compared to the number detected in the invasive component.

We also compared the frequencies of tumor-specific variants between DCIS and adjacent IBC, as shown in Fig. 2. In patient 1, the frequency of tumor-specific variants was higher in the invasive component as compared to the DCIS component, which could not be explained by a difference in tumor cell percentage. This trend was also seen in patient 2, although less tumor-specific variants were detected as compared to patient 1. In patient 3, there was no consistent pattern with respect to differences in the distribution of tumor-specific variant percentages between the two components. For patient 4, there were only four overlapping tumor-specific variants between the invasive component and the in situ component. This patient showed a higher frequency of tumor-specific variants in DCIS as compared to IBC for three out of the four mutations.

**Fig. 2:**
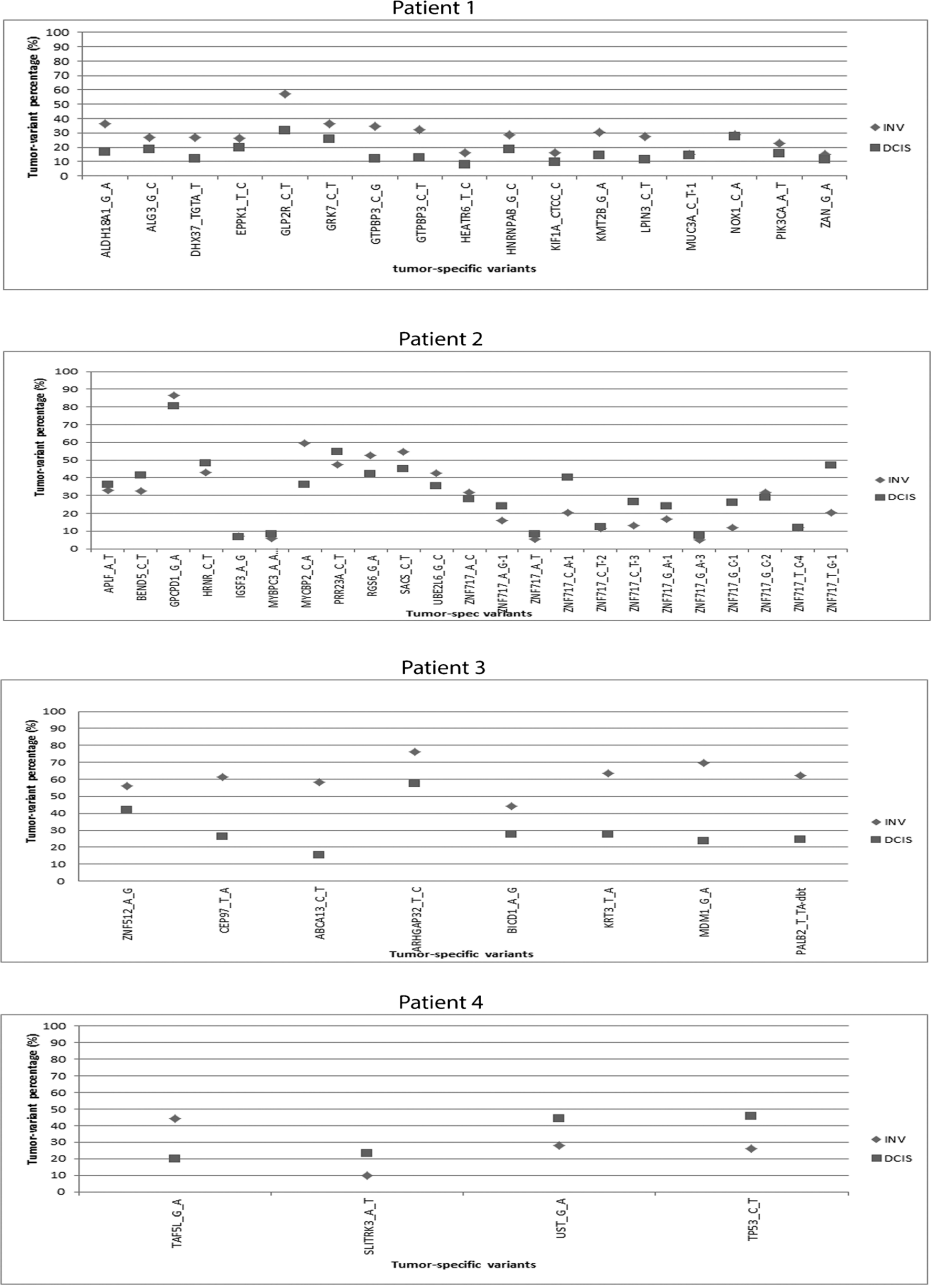
Differences in tumor specific-variant percentages between DCIS (square) and adjacent IBC (rhomb) in all four patients

Taken together, our analyses confirmed a high genomic resemblance between synchronous DCIS and IBC; more than half (52 out of 92) of the mutations identified in the invasive component were also detected in the adjacent in situ component. However, a proportion of mutations (40 out of 92) identified in IBC were not detected in the adjacent DCIS component. In addition, in a subset of patients, the frequencies of the mutations seemed to be higher in the invasive component as compared to DCIS, which could not be explained by the tumor cell percentages in the analyses.

It is important to note that these findings are based on a small number of patients and should be considered as a generated hypothesis only. Besides, our study has several other limitations. First of all, we used two different platforms of massive parallel sequencing. At the validation stage, major differences were observed between these two platforms, due to unreadable sequence regions by Ion Torrent, an insufficient number of reads and false-positive tumor-specific variants (validated in normal tissue by Ion Torrent sequencing). The latter might be the result of a lower sequence depth of whole exome sequencing as compared to Ion Torrent sequencing. In addition, a substantial proportion of variants detected in FF tissue of IBC using Ion Torrent sequencing could not be detected in FFPE tissue of the same tumor, which could be due to tumor heterogeneity. Another limitation of this study is the lack of whole exome data for DCIS, due to lack of available FF tissue of DCIS. Therefore, we could only perform a one-way evaluation of genetic alterations between synchronous DCIS and IBC; genetic alterations restricted to the DCIS component could not be evaluated. At last, we only included ER positive/HER2-negative breast cancer.

In conclusion, we reported overlapping mutations between synchronous DCIS and IBC (with differences regarding the frequencies of mutations between both components), combined with the presence of invasive-specific mutations, which support the theory that DCIS progression could be driven by the selection of subclones. This knowledge might facilitate future studies regarding potential progression markers and novel therapeutic targets in order to establish a more effective personalized treatment for patients with DCIS.

## References

[1] Hernandez L, Wilkerson PM, Lambros MB, Campion-Flora A, Rodrigues DN, Gauthier A, Cabral C, Pawar V, Mackay A, A'Hern R, Marchio C, Palacios J, Natrajan R, Weigelt B, Reis-Filho JS (2012). Genomic and mutational profiling of ductal carcinomas in situ and matched adjacent invasive breast cancers reveals intra-tumour genetic heterogeneity and clonal selection. J Pathol.

[2] Heselmeyer-Haddad K, Berroa Garcia LY, Bradley A, Ortiz-Melendez C, Lee WJ, Christensen R, Prindiville SA, Calzone KA, Soballe PW, Hu Y, Chowdhury SA, Schwartz R, Schaffer AA, Ried T (2012). Single-cell genetic analysis of ductal carcinoma in situ and invasive breast cancer reveals enormous tumor heterogeneity yet conserved genomic imbalances and gain of MYC during progression. Am J Pathol.

[3] Cowell CF, Weigelt B, Sakr RA, Ng CK, Hicks J, King TA, Reis-Filho JS (2013). Progression from ductal carcinoma in situ to invasive breast cancer: revisited. Mol Oncol.

[4] Casasent AK, Edgerton M, Navin NE (2017). Genome evolution in ductal carcinoma in situ: invasion of the clones. J Pathol.

[5] Turner NC, Reis-Filho JS (2012). Genetic heterogeneity and cancer drug resistance. Lancet Oncol.

[6] Yap TA, Gerlinger M, Futreal PA, Pusztai L, Swanton C (2012). Intratumor heterogeneity: seeing the wood for the trees. Sci Transl Med.

[7] Casasent AK, Schalck A, Gao R, Sei E, Long A, Pangburn W, Casasent T, Meric-Bernstam F, Edgerton ME, Navin NE (2018). Multiclonal invasion in breast tumors identified by topographic single cell sequencing. Cell.

[8] Li H, Durbin R (2009). Fast and accurate short read alignment with burrows-wheeler transform. Bioinformatics.

[9] McKenna A, Hanna M, Banks E, Sivachenko A, Cibulskis K, Kernytsky A, Garimella K, Altshuler D, Gabriel S, Daly M, DePristo MA (2010). The genome analysis toolkit: a MapReduce framework for analyzing next-generation DNA sequencing data. Genome Res.

[10] Goecks J, Nekrutenko A, Taylor J, Galaxy T (2010). Galaxy: a comprehensive approach for supporting accessible, reproducible, and transparent computational research in the life sciences. Genome Biol.

